# Some Peculiarities of Oxyphosphates

**Published:** 1895-03

**Authors:** W. V. B. Ames

**Affiliations:** Chicago, Ill


### Some Peculiarities of Oxyphosphates.

A Talk given at the Ohio State Dental Society, December 6, 1894.

BY W. V. B. AMES, CHICAGO, ILL.

MR. PRESIDENT: I did not understand until I came here that I was on the program for a paper. I think I wrote Dr. Callahan how much pleasure it would afford me to be here, and that I would show some peculiarities of oxyphosphates, and I was put on the program for a paper. I do not care to take up the time of the association since you have in store the valuable matter of Dr. Molyneaux and others. I have been doing a great deal of experimenting with oxyphosphate for several years, but I will only call your attention to what I have been able to produce in the way of a phosphoric acid solution which is crystallized in such a form that it can be used as so much liquid and remedies the difficulty which we so often experience from having the liquid of a cement partially crystallized before it is used up. By reason of the form of crystal in my material there is a pasty consistency which is uniform throughout and will not undergo any change from long keeping. I have here also specimens of hardened cement which show greater density and closer texture than we have been familiar within these materials.

DR. H. A. SMITH: How is it as to solubility in the saliva?

DR. AMES: I have seen it hold contour for two years in an approximal cavity in a way that I never have in other materials of this kind.

DR. ——: What dissolves the phosphates?

DR. AMES: When an oxyphosphate dissolves it is either because there is not proper union or crystallization, or there is too much soluble alkaline phosphate cement contained in the mass. Of course the surface exposed to mastication will, with most cements, be worn away; but I believe that a great deal of failure at the cervical margin that is blamed upon the cement is in a large majority of cases the result of faulty manipulation of an undesirable cement. A cement has been used which will adhere tenaciously to a dry surface, but not at all to one that is slightly moist. The cervical margin may have been well prepared and made fairly dry, but if there is a trace of moisture, the cement will be very prone to draw away during the packing or the trimming of the filling. Then of necessity there is failure at the cervical margin. The cements containing soluble alkaline phosphate are the ones which are most apt to behave in this way, therefore they are not best adapted for filling but rather for crown setting. I do not believe that any one cement can be used to advantage for all purposes. We need cement of one quality for filling and of another for setting crowns.

According to my belief and observation, a cement that best answers the purpose of filling is one in the composition of which there are no soluble alkalies. Such a cement mixed as stiff as can be for filling, incorporating as much powder as is practicable, will still have a slight acid taste while plastic, but no taste after proper crystallization. Such a cement mixed thin enough for crown setting would be in improper condition because enough acid would probably dissolve out to leave a friable mass. When we need extreme plasticity we had better use a cement containing alkaline phosphates which gives this plasticity as well as rendering the cement less irritant which is desirable in crown setting and for cavity lining usually. Such a cement placed under a metallic filling or within a crown, can, I believe, be relied upon; whereas, if used for filling purposes independently it would be unreliable.

Dr. Bethel: What is your method for mixing cements?

Dr. Ames: I believe in mixing more energetically than many do. I want a large stiff spatula and large mixing surface. There is a spatula made by the Boston Dental Mfg. Co., their number seven which suits me better than any other which I can buy. As I use my filling cement I mix it as stiff as I can upon the slab, adding the powder gradually and rubbing energetically, gather it up and knead it between the thumb and finger vigorously, incorporating more powder as long as the mass tends to become soft and sticky. After it ceases to take up powder readily there will be sufficient plasticity for packing. In this way I got the best results, getting such density of material as you will see in the specimens passed about. The best of the foreign cements can be worked in this way. There has been a sort of controversy as to whether we should mix cement on a chilled slab or not. Dr. Knapp, of New Orleans, once took exceptions to the statement of Dr. Evans, of New York, that there was an advantage to be gained by mixing the cement on a bottle filled with cold water. Now while this is a good practice in New York during the major portion of the year, it is a practice not well adapted to the atmospheric conditions prevailing in New Orleans where the condensation of moisture would be such as to seriously damage the cement. There is no doubt but that here in the north during the winter months when the air is comparatively free from moisture, we can use such a chilled surface to advantage and do better cement operations than during the summer months when the use of the chilled slab is not practicable.

Dr. ——: What cement do you use?

Dr. Ames: In my practice I use almost entirely that which I produce myself. For crown setting and all work where a non-irritant cement is called for, I use what I have been able to produce, which resembles more nearly the Excelsior, of Ash & Sons than any other. The main difference depends upon my powder, which gives a glossy and close texture. For filling I use about the same powder with the pasty acid, which has been exhibited.

Dr. Taft: This is a subject of much interest, and I think we all realize that a good plastic filling is very desirable. We find a preparation of this kind behaving differently in different cases, and I am hardly able to discover whether that is the result of the material itself—it is at least in some cases—or how much it is dependent on the solvent power of the saliva in different mouths. You find fillings often times that have been for many years in the mouth and seem to be as perfect as when put in. Not long ago I saw a filling in a proximate cavity in an incisor, put in several years ago and there was no wear at all. In others, it wears out in a short time. Frequently when put into a proximate cavity, when it ls under the gum margin, how often it is underminded and the cement worn out at the cervical portion of the cavity and the rest remains in good condition. That could not be on account of the peculiar condition of the cement, but because a solvent is secreted by the mucous membrane. It may be it was not properly introduced.

Dr. ——: What is it that destroys it there?

Dr. Taft: It is an acid of the saliva. I have long desired to find the cement that will serve this purpose satisfactorily and have not been able to find it. It is a line that ought to be investigated. Investigation should be made in regard to the quality of material and the reliance that can be put upon it for the various purposes for which it is used. In the mouths of some persons cement will not wear any considerable length of time exposed to the secretions of the mouth, the mucous is a more active solvent than the saliva proper. A desirable point is to gain a knowledge of the best material that will be reliable and its behavior be uniform. Different cements behave differently under different circumstances. Sometimes they are easily manipulated and sometimes quite otherwise. These variations I am not able to clearly account for.

I suppose the experiments of Dr. Ames are with a view of settling these questions. I think he can help us settle them. He has experimented more extensively than anybody else.

Dr. Sillito to Ames: Do the liquids crystallize on account of evaporation?

Dr. Ames: The matter of crystallization of cement liquids is not necessarily a matter of absorption or giving up or moisture. A certain solution may remain in the liquid state for a considerable time and then begin to crystallize from the effects even of a sudden jar, but more likely because of there being placed within it some nucleus about which the crystals will readily form. Such a nucleus is easily introduced by means of the spatula or whatever is used in transferring the liquid from the bottle to the mixing surface. With many of the liquids crystallization can be avoided by dropping from the bottle instead of inserting spatulas, etc., within the bottle. My main object in speaking to this extent, Mr. President, is not to advertise my materials as it may seem, but to impress upon the members my conviction that a great deal of good can be accomplished by the use of the proper cements in a differential way. If a man tells me he does not believe in cements for filling purposes, and then tells me that he uses certain materials I might mention, I say of course they will not stand. They might do for setting crowns, but I would not make a filling of them. If you use such a cement as the Harvard, or Dirigo, or Globe, or Concrete, or Eisfelder's, a very useful filling can be made.

Dr. Smith asks whether an acid or alkaline condition is more destructive to cements. I can not venture to say what the result would be with faulty material. I can only say that I believe that with material properly compounded and properly manipulated, there will be no solution, but only more or less wearing away of the most exposed surface.

Dr. Taft, asked Dr. Ames to mention the names of three or four cements that he considered the best.

Dr. Ames: I mentioned that the cement which I use for crown setting resembles the Excelsior of Ash & Sons, which I regard as very satisfactory for this purpose. It is well thought of by the men in New York and Philadelphia whom we best know. I have mentioned the Harvard, Dirigo, Concrete, Globe, Eisfelder's, and I might include some other German preparations possibly for satisfactory filling cements. What I use myself I term Ames' metalloid, which if worked as I have recommended, gives satisfaction. After mixing it as I advised, and packing it into the cavity, I work more powder into the surface as it is being shaped up. As the filling has the desired contour, rub the surface down with a pellet of cotton or some such material which is *very slightly* moistened, as you get by moistening a pellet and squeezing between dry surfaces of napkin. By means of this slightly moistened pellet the surface can be smoothed, and this slight moisture is an advantage with the cement which I use for fillings. If I have plenty of time to allow the filling to harden while I am making some other operation, I would work it down to the proper contour as nearly as possible, and use the moistened pellet just before using the rubber dam. I use a matrix wherever practicable, so as to facilitate the packing and finishing.

Dr. Sillito: I notice that the Dirigo cement is more lasting if you allow the central portion of the filling to bulge.

Dr. Ames: I have not used it enough to learn that. I will say in this connection that there is in the Dirigo a considerable portion of an antiseptic which is unmistakably hydronapthol. I do not believe that any advantage is gained by incorporating such an antiseptic with a cement. I believe that it detracts from the density and wearing qualities, and does not exert any valuable antiseptic influence.

Dr. ——: There is one cement you have not mentioned, namely, Brittons:

Dr. Ames: I would rather use Excelsior than Brittons. I believe it is equally servicable and much pleasanter to manipulate.

